# Supplementary material for: Modeling glioblastoma heterogeneity as a dynamic network of cell states
Source: Mol Syst Biol. 2021 Sep 16;17(9):e10105. doi: 10.15252/msb.202010105 (PMC8444284; doi:10.15252/msb.202010105)
Supplement: Supplementary file 6 — Source Data for Figure 5 [file MSB-17-e10105-s004.zip › Figure5A_sourcedata/GSEA_3017/nosigdb_stateA.GseaPreranked.1622795026485/RICHARDS_NOWAKOWSKI_SCIENCE_2017_IPC_DIV2_UPREG.html]

Details for gene set RICHARDS\_NOWAKOWSKI\_SCIENCE\_2017\_IPC\_DIV2\_UPREG[GSEA]

|  || Dataset | state53017 |
| Phenotype | NoPhenotypeAvailable |
| Upregulated in class | na\_pos |
| GeneSet | RICHARDS\_NOWAKOWSKI\_SCIENCE\_2017\_IPC\_DIV2\_UPREG |
| Enrichment Score (ES) | 0.36217812 |
| Normalized Enrichment Score (NES) | 3.2233782 |
| Nominal p-value | 0.0 |
| FDR q-value | 0.0 |
| FWER p-Value | 0.0 |
Table: GSEA Results Summary

  

Fig 1: Enrichment plot: RICHARDS\_NOWAKOWSKI\_SCIENCE\_2017\_IPC\_DIV2\_UPREG      
 Profile of the Running ES Score & Positions of GeneSet Members on the Rank Ordered List

  

| PROBE | GENE SYMBOL | GENE\_TITLE | RANK IN GENE LIST | RANK METRIC SCORE | RUNNING ES | CORE ENRICHMENT || 1 | FABP5 |  |  | 0 | 1.759 | 0.0216 | Yes |
| 2 | CDK1 |  |  | 3 | 1.006 | 0.0313 | Yes |
| 3 | MKI67 |  |  | 4 | 0.926 | 0.0427 | Yes |
| 4 | KIF11 |  |  | 6 | 0.870 | 0.0521 | Yes |
| 5 | UBE2C |  |  | 7 | 0.793 | 0.0618 | Yes |
| 6 | MCM10 |  |  | 9 | 0.752 | 0.0697 | Yes |
| 7 | KIF20B |  |  | 10 | 0.749 | 0.0789 | Yes |
| 8 | BUB3 |  |  | 12 | 0.740 | 0.0867 | Yes |
| 9 | KPNA2 |  |  | 20 | 0.667 | 0.0860 | Yes |
| 10 | HIST1H4C |  |  | 21 | 0.659 | 0.0940 | Yes |
| 11 | RPS24 |  |  | 23 | 0.649 | 0.1007 | Yes |
| 12 | CA12 |  |  | 24 | 0.645 | 0.1086 | Yes |
| 13 | ASPM |  |  | 27 | 0.639 | 0.1139 | Yes |
| 14 | DNAJC9 |  |  | 34 | 0.615 | 0.1138 | Yes |
| 15 | PGAM1 |  |  | 35 | 0.608 | 0.1212 | Yes |
| 16 | PLCB1 |  |  | 36 | 0.604 | 0.1286 | Yes |
| 17 | CKS2 |  |  | 40 | 0.587 | 0.1320 | Yes |
| 18 | NCAPG |  |  | 44 | 0.556 | 0.1350 | Yes |
| 19 | HJURP |  |  | 47 | 0.539 | 0.1390 | Yes |
| 20 | CKS1B |  |  | 50 | 0.530 | 0.1430 | Yes |
| 21 | GLRX3 |  |  | 51 | 0.529 | 0.1495 | Yes |
| 22 | DBF4 |  |  | 55 | 0.514 | 0.1519 | Yes |
| 23 | SMC3 |  |  | 58 | 0.504 | 0.1555 | Yes |
| 24 | KIF14 |  |  | 59 | 0.503 | 0.1617 | Yes |
| 25 | MASTL |  |  | 60 | 0.499 | 0.1678 | Yes |
| 26 | CENPE |  |  | 62 | 0.496 | 0.1726 | Yes |
| 27 | MIS18BP1 |  |  | 64 | 0.495 | 0.1774 | Yes |
| 28 | CCNA2 |  |  | 65 | 0.495 | 0.1835 | Yes |
| 29 | TPX2 |  |  | 66 | 0.488 | 0.1895 | Yes |
| 30 | RPP30 |  |  | 68 | 0.486 | 0.1941 | Yes |
| 31 | MYBL2 |  |  | 69 | 0.486 | 0.2001 | Yes |
| 32 | HNRNPF |  |  | 70 | 0.485 | 0.2061 | Yes |
| 33 | MMP16 |  |  | 74 | 0.477 | 0.2081 | Yes |
| 34 | DLGAP5 |  |  | 76 | 0.475 | 0.2126 | Yes |
| 35 | HIST1H1B |  |  | 77 | 0.475 | 0.2184 | Yes |
| 36 | VRK1 |  |  | 82 | 0.470 | 0.2191 | Yes |
| 37 | UBE2T |  |  | 83 | 0.470 | 0.2248 | Yes |
| 38 | DUT |  |  | 87 | 0.466 | 0.2267 | Yes |
| 39 | PLK1 |  |  | 89 | 0.464 | 0.2311 | Yes |
| 40 | CENPF |  |  | 94 | 0.461 | 0.2317 | Yes |
| 41 | TOP2A |  |  | 95 | 0.457 | 0.2373 | Yes |
| 42 | HIST1H1D |  |  | 98 | 0.456 | 0.2403 | Yes |
| 43 | PRDX3 |  |  | 100 | 0.453 | 0.2446 | Yes |
| 44 | PRTFDC1 |  |  | 104 | 0.448 | 0.2462 | Yes |
| 45 | RBM17 |  |  | 105 | 0.447 | 0.2517 | Yes |
| 46 | ZWINT |  |  | 106 | 0.447 | 0.2572 | Yes |
| 47 | TGIF1 |  |  | 107 | 0.447 | 0.2627 | Yes |
| 48 | AURKB |  |  | 108 | 0.446 | 0.2681 | Yes |
| 49 | ZEB1 |  |  | 109 | 0.446 | 0.2736 | Yes |
| 50 | CDCA8 |  |  | 115 | 0.437 | 0.2726 | Yes |
| 51 | KIF23 |  |  | 116 | 0.436 | 0.2779 | Yes |
| 52 | NSMCE4A |  |  | 117 | 0.435 | 0.2832 | Yes |
| 53 | MELK |  |  | 123 | 0.427 | 0.2821 | Yes |
| 54 | BUB1B |  |  | 129 | 0.421 | 0.2808 | Yes |
| 55 | HELLS |  |  | 135 | 0.417 | 0.2796 | Yes |
| 56 | RTKN2 |  |  | 141 | 0.413 | 0.2782 | Yes |
| 57 | CTBP2 |  |  | 142 | 0.413 | 0.2833 | Yes |
| 58 | GTSE1 |  |  | 146 | 0.411 | 0.2845 | Yes |
| 59 | CCNB2 |  |  | 147 | 0.411 | 0.2895 | Yes |
| 60 | ANLN |  |  | 152 | 0.407 | 0.2894 | Yes |
| 61 | MAD2L1 |  |  | 153 | 0.406 | 0.2944 | Yes |
| 62 | LINC01224 |  |  | 154 | 0.406 | 0.2994 | Yes |
| 63 | PBX3 |  |  | 157 | 0.404 | 0.3018 | Yes |
| 64 | EIF4EBP2 |  |  | 158 | 0.403 | 0.3067 | Yes |
| 65 | KIFC1 |  |  | 160 | 0.402 | 0.3104 | Yes |
| 66 | ARHGAP11A |  |  | 162 | 0.399 | 0.3140 | Yes |
| 67 | GNG4 |  |  | 164 | 0.398 | 0.3176 | Yes |
| 68 | KIF2C |  |  | 165 | 0.397 | 0.3225 | Yes |
| 69 | MCM7 |  |  | 171 | 0.394 | 0.3209 | Yes |
| 70 | NDC80 |  |  | 173 | 0.393 | 0.3244 | Yes |
| 71 | HNRNPH3 |  |  | 185 | 0.387 | 0.3151 | Yes |
| 72 | BUB1 |  |  | 188 | 0.384 | 0.3173 | Yes |
| 73 | CMSS1 |  |  | 191 | 0.381 | 0.3194 | Yes |
| 74 | KIF22 |  |  | 197 | 0.377 | 0.3176 | Yes |
| 75 | CCNY |  |  | 199 | 0.375 | 0.3209 | Yes |
| 76 | CDCA3 |  |  | 203 | 0.372 | 0.3217 | Yes |
| 77 | SUV39H2 |  |  | 205 | 0.371 | 0.3249 | Yes |
| 78 | PTMA |  |  | 206 | 0.371 | 0.3295 | Yes |
| 79 | SMC4 |  |  | 217 | 0.367 | 0.3212 | Yes |
| 80 | SPC25 |  |  | 226 | 0.363 | 0.3154 | Yes |
| 81 | INCENP |  |  | 227 | 0.362 | 0.3199 | Yes |
| 82 | NCAPH |  |  | 232 | 0.359 | 0.3191 | Yes |
| 83 | H2AFZ |  |  | 245 | 0.351 | 0.3081 | Yes |
| 84 | PPIF |  |  | 248 | 0.350 | 0.3098 | Yes |
| 85 | ITGB1 |  |  | 256 | 0.346 | 0.3051 | Yes |
| 86 | FEN1 |  |  | 259 | 0.343 | 0.3068 | Yes |
| 87 | TTK |  |  | 262 | 0.340 | 0.3084 | Yes |
| 88 | TUBB |  |  | 264 | 0.340 | 0.3113 | Yes |
| 89 | SLC25A5 |  |  | 266 | 0.340 | 0.3142 | Yes |
| 90 | CENPJ |  |  | 270 | 0.339 | 0.3145 | Yes |
| 91 | LRR1 |  |  | 273 | 0.337 | 0.3161 | Yes |
| 92 | CLSPN |  |  | 274 | 0.336 | 0.3202 | Yes |
| 93 | DEPDC1B |  |  | 275 | 0.335 | 0.3243 | Yes |
| 94 | ESCO2 |  |  | 282 | 0.333 | 0.3207 | Yes |
| 95 | ORC6 |  |  | 283 | 0.333 | 0.3248 | Yes |
| 96 | C21orf58 |  |  | 294 | 0.328 | 0.3160 | Yes |
| 97 | GGCT |  |  | 298 | 0.326 | 0.3162 | Yes |
| 98 | MND1 |  |  | 304 | 0.324 | 0.3138 | Yes |
| 99 | RAD18 |  |  | 307 | 0.324 | 0.3152 | Yes |
| 100 | BRCA2 |  |  | 308 | 0.323 | 0.3191 | Yes |
| 101 | CENPN |  |  | 310 | 0.323 | 0.3218 | Yes |
| 102 | FANCI |  |  | 319 | 0.319 | 0.3155 | Yes |
| 103 | DNA2 |  |  | 321 | 0.318 | 0.3181 | Yes |
| 104 | RFC3 |  |  | 337 | 0.314 | 0.3028 | Yes |
| 105 | CDCA5 |  |  | 341 | 0.312 | 0.3028 | Yes |
| 106 | CLIC1 |  |  | 342 | 0.311 | 0.3066 | Yes |
| 107 | CHD7 |  |  | 343 | 0.311 | 0.3104 | Yes |
| 108 | PRC1 |  |  | 350 | 0.310 | 0.3065 | Yes |
| 109 | RPS3 |  |  | 356 | 0.307 | 0.3039 | Yes |
| 110 | ANP32B |  |  | 357 | 0.307 | 0.3077 | Yes |
| 111 | SLC20A1 |  |  | 361 | 0.306 | 0.3076 | Yes |
| 112 | ECT2 |  |  | 365 | 0.305 | 0.3075 | Yes |
| 113 | CCNF |  |  | 366 | 0.305 | 0.3112 | Yes |
| 114 | TTF2 |  |  | 367 | 0.305 | 0.3149 | Yes |
| 115 | CDCA2 |  |  | 369 | 0.304 | 0.3174 | Yes |
| 116 | BRCA1 |  |  | 373 | 0.302 | 0.3173 | Yes |
| 117 | CKAP2 |  |  | 379 | 0.301 | 0.3146 | Yes |
| 118 | RPA3 |  |  | 381 | 0.300 | 0.3170 | Yes |
| 119 | CSE1L |  |  | 382 | 0.300 | 0.3206 | Yes |
| 120 | RRM2 |  |  | 383 | 0.300 | 0.3243 | Yes |
| 121 | XRCC2 |  |  | 384 | 0.300 | 0.3280 | Yes |
| 122 | CENPW |  |  | 386 | 0.299 | 0.3304 | Yes |
| 123 | TEX30 |  |  | 388 | 0.298 | 0.3328 | Yes |
| 124 | CDC6 |  |  | 391 | 0.297 | 0.3338 | Yes |
| 125 | PHF19 |  |  | 393 | 0.297 | 0.3362 | Yes |
| 126 | SAAL1 |  |  | 398 | 0.295 | 0.3347 | Yes |
| 127 | RACGAP1 |  |  | 399 | 0.293 | 0.3383 | Yes |
| 128 | PA2G4 |  |  | 402 | 0.293 | 0.3393 | Yes |
| 129 | HMGB1 |  |  | 403 | 0.292 | 0.3429 | Yes |
| 130 | NME4 |  |  | 404 | 0.291 | 0.3465 | Yes |
| 131 | CREB5 |  |  | 407 | 0.291 | 0.3475 | Yes |
| 132 | CCT5 |  |  | 409 | 0.290 | 0.3498 | Yes |
| 133 | NFIA |  |  | 413 | 0.289 | 0.3495 | Yes |
| 134 | DCP2 |  |  | 416 | 0.289 | 0.3505 | Yes |
| 135 | CSGALNACT2 |  |  | 419 | 0.288 | 0.3514 | Yes |
| 136 | HMGB2 |  |  | 424 | 0.288 | 0.3499 | Yes |
| 137 | NUSAP1 |  |  | 430 | 0.286 | 0.3470 | Yes |
| 138 | NUF2 |  |  | 434 | 0.285 | 0.3466 | Yes |
| 139 | MIS18A |  |  | 439 | 0.284 | 0.3450 | Yes |
| 140 | NCAPD2 |  |  | 440 | 0.283 | 0.3485 | Yes |
| 141 | LMNB1 |  |  | 442 | 0.282 | 0.3506 | Yes |
| 142 | CDC25C |  |  | 443 | 0.282 | 0.3541 | Yes |
| 143 | CDC45 |  |  | 446 | 0.282 | 0.3550 | Yes |
| 144 | KIF20A |  |  | 447 | 0.281 | 0.3585 | Yes |
| 145 | FBL |  |  | 451 | 0.281 | 0.3581 | Yes |
| 146 | ANP32E |  |  | 453 | 0.280 | 0.3602 | Yes |
| 147 | GINS1 |  |  | 462 | 0.279 | 0.3534 | Yes |
| 148 | SMS |  |  | 463 | 0.278 | 0.3568 | Yes |
| 149 | MNS1 |  |  | 464 | 0.278 | 0.3602 | Yes |
| 150 | ATAD2 |  |  | 470 | 0.275 | 0.3572 | Yes |
| 151 | EZH2 |  |  | 471 | 0.275 | 0.3606 | Yes |
| 152 | SKA1 |  |  | 475 | 0.274 | 0.3601 | Yes |
| 153 | SHCBP1 |  |  | 477 | 0.274 | 0.3622 | Yes |
| 154 | NHLRC2 |  |  | 488 | 0.272 | 0.3527 | No |
| 155 | BLM |  |  | 490 | 0.272 | 0.3548 | No |
| 156 | PSAT1 |  |  | 491 | 0.271 | 0.3581 | No |
| 157 | REEP3 |  |  | 496 | 0.271 | 0.3563 | No |
| 158 | KIF4A |  |  | 504 | 0.269 | 0.3507 | No |
| 159 | UCK2 |  |  | 508 | 0.269 | 0.3501 | No |
| 160 | HAUS8 |  |  | 518 | 0.267 | 0.3419 | No |
| 161 | ZNF90 |  |  | 520 | 0.266 | 0.3439 | No |
| 162 | RCC1 |  |  | 528 | 0.265 | 0.3382 | No |
| 163 | SKA3 |  |  | 531 | 0.264 | 0.3389 | No |
| 164 | ALYREF |  |  | 535 | 0.264 | 0.3383 | No |
| 165 | RANBP1 |  |  | 536 | 0.263 | 0.3415 | No |
| 166 | KIF15 |  |  | 538 | 0.263 | 0.3434 | No |
| 167 | CDKN3 |  |  | 539 | 0.263 | 0.3466 | No |
| 168 | SRSF2 |  |  | 542 | 0.262 | 0.3473 | No |
| 169 | STIL |  |  | 545 | 0.261 | 0.3479 | No |
| 170 | CENPO |  |  | 549 | 0.260 | 0.3473 | No |
| 171 | FBXO5 |  |  | 557 | 0.258 | 0.3415 | No |
| 172 | DDX39A |  |  | 562 | 0.258 | 0.3396 | No |
| 173 | GNB4 |  |  | 563 | 0.258 | 0.3427 | No |
| 174 | ZMIZ1 |  |  | 573 | 0.255 | 0.3343 | No |
| 175 | TMPO |  |  | 584 | 0.253 | 0.3246 | No |
| 176 | ZNF714 |  |  | 594 | 0.251 | 0.3162 | No |
| 177 | FAM200B |  |  | 598 | -0.250 | 0.3154 | No |
| 178 | SYPL1 |  |  | 608 | -0.255 | 0.3071 | No |
| 179 | FAM111A |  |  | 618 | -0.259 | 0.2987 | No |
| 180 | DAG1 |  |  | 622 | -0.260 | 0.2981 | No |
| 181 | TMTC2 |  |  | 633 | -0.263 | 0.2885 | No |
| 182 | SHROOM3 |  |  | 639 | -0.264 | 0.2854 | No |
| 183 | MAGI1 |  |  | 642 | -0.266 | 0.2861 | No |
| 184 | EML1 |  |  | 649 | -0.269 | 0.2817 | No |
| 185 | CKB |  |  | 676 | -0.287 | 0.2520 | No |
| 186 | ZFHX4 |  |  | 700 | -0.301 | 0.2262 | No |
| 187 | ELOVL2 |  |  | 735 | -0.329 | 0.1868 | No |
| 188 | LRRCC1 |  |  | 746 | -0.341 | 0.1782 | No |
| 189 | MYO6 |  |  | 750 | -0.343 | 0.1786 | No |
| 190 | DENND5B |  |  | 757 | -0.347 | 0.1751 | No |
| 191 | WSCD1 |  |  | 761 | -0.351 | 0.1756 | No |
| 192 | CUX1 |  |  | 775 | -0.364 | 0.1634 | No |
| 193 | SETBP1 |  |  | 779 | -0.368 | 0.1641 | No |
| 194 | NKAIN3 |  |  | 808 | -0.389 | 0.1331 | No |
| 195 | SSX2IP |  |  | 810 | -0.392 | 0.1366 | No |
| 196 | TMSB4X |  |  | 850 | -0.443 | 0.0922 | No |
| 197 | TMEM47 |  |  | 855 | -0.447 | 0.0925 | No |
| 198 | TFDP2 |  |  | 858 | -0.448 | 0.0955 | No |
| 199 | EZR |  |  | 865 | -0.464 | 0.0935 | No |
| 200 | LZTS1 |  |  | 866 | -0.467 | 0.0992 | No |
| 201 | ZNF106 |  |  | 873 | -0.483 | 0.0975 | No |
| 202 | DAAM1 |  |  | 885 | -0.516 | 0.0897 | No |
| 203 | GPC6 |  |  | 896 | -0.540 | 0.0835 | No |
| 204 | SDC2 |  |  | 908 | -0.568 | 0.0764 | No |
| 205 | CSRP2 |  |  | 960 | -0.877 | 0.0220 | No |
| 206 | CDK6 |  |  | 966 | -0.920 | 0.0269 | No |
Table: GSEA details [plain text format]

  

Fig 2: RICHARDS\_NOWAKOWSKI\_SCIENCE\_2017\_IPC\_DIV2\_UPREG: Random ES distribution      
 Gene set null distribution of ES for **RICHARDS\_NOWAKOWSKI\_SCIENCE\_2017\_IPC\_DIV2\_UPREG**
